# 

**Published:** 1876-06

**Authors:** 


Vol. XXXIII.
Number 6.

June, 1876.

NEW SERIES.
Vol. I.
Number 6.

THE

### CHICAGO MEDICAL Journal and Examiner

PUBLISHED MONTHLY.

**EDITOR:**
WILLIAM H. BYFORD, A.M., M.D.

**ASSOCIATE EDITORS:**

JAMES H. ETHERIDGE, M.D. | JAS. NEVINS HYDE, A.M., M.D.

NORMAN BRIDGE, M.D. | FERD. C. HOTZ, M.D.

Published under the auspices of the

*CHICAGO MEDICAL PRESS ASSOCIATION.*

**TERMS–FOUR DOLLARS PER ANNUM, IN ADVANCE.
POSTAGE FREE.**

CHICAGO:
W. B. KEEN, COOKE & CO., Publishers,
Nos. 113 and 115 State Street.

